# Cutaneous pharmacokinetics of a volatile drug post-application to the skin

**DOI:** 10.1007/s13346-025-01907-8

**Published:** 2025-06-26

**Authors:** Andrea Pensado, Panagiota Zarmpi, Jane White, Annette L. Bunge, Richard H. Guy, M. Begoña Delgado-Charro

**Affiliations:** 1 Department of Life Sciences, University of Bath, Claverton Down, Bath, BA2 7AY UK; 2 Present address: Center for Research in Molecular Medicine & Chronic Diseases (CiMUS), Universidade de Santiago de Compostela, Santiago de Compostela, 15782 Spain; 3 Present address: School of Chemistry and Chemical Engineering, University of Surrey, Guildford, GU2 7XH UK; 4 Department of Mathematical Sciences, University of Bath, Bath, BA2 7AY UK; 5 Department of Chemical & Biological Engineering, Colorado School of Mines, Golden, 80401 CO USA

**Keywords:** Methyl salicylate, Topical bioavailability, Volatile drug, Cutaneous pharmacokinetics, Tape stripping

## Abstract

This research explored whether in vitro release and skin permeation tests, combined with in vitro and in vivo stratum corneum (SC) sampling, can quantify the “input rate” of a volatile drug into and through the skin. Two topical methyl salicylate (MeSA) products were studied. Drug release from the formulations across a silicone membrane was similar, with ~ 65% of the drug load being released in 6 h. In vitro porcine skin permeation tests showed that ~ 50% of the dose crossed the skin in 24 h from both products. In SC sampling experiments, the mass of MeSA in the SC at one ‘uptake’ and three ‘clearance’ time points was measured in vitro and in vivo (in humans). The MeSA quantity taken up into the SC in vitro was > 10-fold higher than that in vivo. The first-order rate constants describing clearance from the SC were calculated and found to be similar, in vitro and in vivo, for both formulations. Mass balance revealed that about one-third of the applied drug may have been lost by evaporation in vitro from the skin surface during 2 h of clearance. The results show that the cutaneous pharmacokinetics of MeSA are proportional to the amount of drug in the two formulations and that the complementary use of in vitro release and skin penetration tests with the SC sampling technique are valid tools with which to assess topical products containing volatile drugs.

**Graphical abstract:**

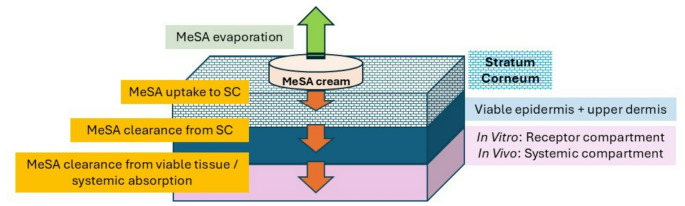

**Supplementary Information:**

The online version contains supplementary material available at 10.1007/s13346-025-01907-8.

## Introduction

The treatment of localised skin diseases with topically applied drug formulations makes sense from both efficacy and safety criteria, i.e., delivery in close proximity to the site where pharmacological action is required while minimising systemic exposure [1]. However, unlike the situation for centrally-acting active pharmaceutical ingredients (APIs), pharmacokinetic (PK) studies for drugs that act locally are challenging due to the conventional definition of bioavailability (BA): *“the rate and extent at which the active species is absorbed from a formulation and becomes available at (or*,* at least*,* near) the site of action”* [2]. In this case, the blood/plasma/serum is not an automatic choice as the surrogate compartment in which to measure ‘rate and extent’ because (a) it is not always clear that drug levels here accurately inform the compound’s disposition in the skin, and/or (b) the levels of API in the systemic compartment may be very difficult or impossible to measure [3–5]. As a result, there is an ongoing effort to identify and validate experimental tools with which to evaluate the cutaneous disposition and PK of topically applied drugs. Much of this work in the recent past has been driven by the closely-related objective of assessing the bioequivalence (BE) between complex drug products intended for application to the skin.

Already, it is clear that focus on this challenge to determine topical BA/BE is yielding progress via the issuance of several product specific guidances by the U.S. Food & Drug Administration (FDA) [6], for example, and the 2024 publication of a guideline on *“quality and equivalence of locally applied*,* locally acting cutaneous products”* from the European Medicines Agency (EMA) [7]. In both instances, there is an evident shift towards the use of one or more experimental tools that alone, or (more typically) in combination can be applied to better assure the quality of topical cutaneous products and to characterise ‘rate and extent’ either in vitro or in vivo or both. In this way, a potential pathway to establish therapeutic efficacy/BA (and hence BE between products) may be identified, either providing important support to clinical endpoint studies or– in the case of BE– relieve the significant manpower and financial burden faced by generic product developers [8–11].

Concerning the methods that have received significant attention over the last decade or more, the in vitro release test (IVRT) is already embedded as a component of product quality characterisation [12, 13]. Equally, the in vitro permeation test (IVPT) is now routinely expected to be used to establish the skin uptake (amount and rate) of the API from a cutaneous product [7, 14]. Stratum corneum (SC) sampling using tape-stripping has long been recognised by the Pharmaceuticals and Medical Devices Agency in Japan [15] and is an approach endorsed by the just-published EMA guideline [7]; for the moment, however, the FDA has not publicly revisited the value of this approach. Collectively, these techniques can deduce the input rate of drugs which is the process by which the API transfers from the SC into the viable tissue [16–18]. Of course, the vasoconstriction assay remains universally accepted as a surrogate method specifically for corticosteroids [7, 19, 20]. Other methods are being closely studied for their ability to measure drug diffusion and content within the skin– of note are confocal Raman spectroscopy and microdialysis/open-flow microperfusion [21–23]– but have yet to gain formal regulatory recognition.

The objective of this work was to investigate whether IVRT, IVPT and SC sampling are suitable methods with which to assess the cutaneous disposition and PK of a locally-acting drug of significant volatility [24]. Clearly, such compounds and their formulations pose additional challenges with respect to the measurement of BA/BE. For this purpose, methyl salicylate (MeSA) in two formulations containing different concentrations of the API and some differences in excipient composition were chosen. While the skin permeation characteristics of MeSA have been considered before [25], the focus of the literature has been primarily on metabolism [26, 27] with little effort devoted to the question of the impact of the drug’s volatility on its cutaneous uptake and disposition.

## Materials and methods

### Materials

Two commercially available methyl salicylate (MeSA) cream products were used: 12.8% MeSA Deep Heat Rub^®^ and 30% MeSA Deep Heat Max^®^ (Mentholatum, East Kilbride, Scotland, UK). Drug content of the formulations was verified. The compositions of the formulations are in Table 1. High-performance liquid chromatography (HPLC) grade methanol (MeOH) and acetic acid (> 99.7% purity) were obtained from VWR (Leicestershire, UK). Water was ultra-pure (Milli-Q) laboratory grade.

**Table 1:** Composition of the methyl salicylate cream formulations. information taken from the patient information leaflets for Deep Heat Rub [28] and Deep Heat Max [29]. Note that according to the Medicines and Healthcare products Regulatory Agency regulations, the Summary of Product Characteristics only provides quantitative composition of active ingredients and of functional excipients

	Deep Heat Rub	Deep Heat Max
Active ingredients	Methyl salicylate, 12.8% w/w Menthol, 5.91% w/w Eucalyptus oil, 1.97% w/w Turpentine oil, 1.47% w/w	Methyl salicylate, 30.0% w/w Menthol, 8.0% w/w
Inactive ingredients	Sodium cetostearyl sulphate Cetostearyl alcohol Propylene glycol Wool fat (Lanolin) Liquid paraffin Water	Sorbitan stearate Polyoxyethylene hexadecyl ether Glyceryl stearate Sodium lauryl sulphate Poloxamer 407 Water

### In vitro release test (IVRT) experiments

The release of MeSA from the two creams was measured across a non-porous silicone membrane (75 μm thickness; Dow Corning 7-4107, Auburn, MI) over 24 h. The membranes were soaked in MeOH/water (30:70) for 0.5 h before mounting in the vertical Franz diffusion cells with an active area of 2.01 cm^2^ (PermeGear, Inc., Bethlehem, USA). Given the published data on MeSA’s aqueous solubility and to ensure that sink conditions were maintained for all experiments [25, 30], the same MeOH/water mixture was used as the receptor phase (7.4 mL). Creams were applied at 5 mg cm^− 2^ using inverted 1.5 mL centrifuge tubes (Eppendorf^®^, Stevenage, UK) and the exact mass of product applied was determined by weight difference [weighing balance (BP 210D, Sartorius Ltd, UK)] between the initial and residual product on the centrifuge tube. The choice of dose was guided by the Patient Information Leaflet and by the need to cover the whole area of the study with a thin layer of product. Post-application, and throughout the experiment, the donor chamber of the diffusion cell (and the sampling port) were covered with Parafilm. Samples (1 mL) of the receptor phase were withdrawn at 0.5, 1, 2, 4, 6 and 24 h, replaced with fresh receptor solution, and the sampling port covered with fresh Parafilm. The diffusion cells were kept in an oven at 32 °C and 40% relative humidity, except for brief excursions when the receptor solution was sampled. Receptor samples were filtered (Sartorius minisart syringe filters, 4 mm diameter, 0.45 μm pore size regenerated cellulose filters (Sartorius AG, Göttingen, Germany)) and the concentration of MeSA in the samples was quantified by HPLC-UV. All experiments were performed in sextuplicate. As a test of MeSA’s permeability through Parafilm, a similar series of experiments were performed (in triplicate) using Parafilm (127 μm thick [31]) as the membrane and withdrawing samples from the receptor phase at 2, 4, 6 and 24 h.

### In vitro skin permeation test (IVPT) experiments

Excised dorsal porcine skin, sourced from a local abattoir and dermatomed (Zimmer dermatome, Dover, DE) to a nominal 750 μm thickness, was used. The skin was stored at -20 °C before use and thawed, at room temperature, prior to the start of each experiment. Visible hairs were carefully clipped away. The skin was mounted in the diffusion cells with the dermal side in contact with the magnetically stirred receptor medium (MeOH/water 30:70). As for the IVRT experiments, the creams were applied for 24 h at 5 mg cm^− 2^ using inverted centrifuge tubes, and the actual mass of product applied was determined by weight difference. Similarly, throughout the experiment, the donor compartment of the diffusion cells remained covered with the same piece of Parafilm, and the diffusion cells were maintained in an oven at 32 °C and 40% relative humidity, except for brief excursions at 1, 2, 4, 6, 8, and 24 h when the receptor solution was sampled and the Parafilm on the sampling port was replaced with a new piece. Aliquots of 1 mL of the receptor were withdrawn and immediately replaced with the same volume of fresh receptor solution. Samples were filtered and the concentration of MeSA therein was quantified by HPLC-UV. All experiments were performed in sextuplicate.

### In vitro stratum corneum and viable tissue sampling

The IVPT procedure described above was repeated in all details except that the experiment duration was reduced to just 2 h. From each cell, a 1-mL sample of the receptor solution at 2 h (but not at 1 h) was reserved for HPLC analysis of permeated MeSA. At 2 h, the donor cell was removed and the residual formulation on the skin surface was cleaned with one dry wipe (Wypall, Kimberly Clark, Kent, UK) followed by cleaning with three successive 70% isopropyl alcohol wipes (Sterets^®^, Molnlycke, Lancashire, UK) applied quickly to minimize drug extraction from the skin. Drug present in the SC was then assessed immediately by sampling this layer of the skin using a tape-stripping procedure that followed closely a published methodology [32]. Briefly, the surface-cleaned skin sample was pinned to a polystyrene board and covered with a polypropylene template with a 2.01 cm^2^ circular opening that left the treated skin area exposed. Adhesive tape strips (2.5 cm × 2.5 cm; 3 M Scotch tape, Gresswell, UK) were applied to the exposed SC, pressed firmly to ensure uniform contact, and rapidly removed to sample the outermost skin layers. The direction of stripping was rotated 90° with each successive tape to minimize directional bias. This process was repeated for a total of 20 tape strips. Tape-strips were extracted in groups by stripping number, typically 1, 2, 3–6, 7–10, 11–15 and 16–20, to ensure that the extraction solvent for each contained a quantifiable amount of MeSA. The drug was then extracted from the tape-strips into 3 mL of extraction solvent (MeOH/acetic acid (1%) 90:10), 0.5 h of sonication, and overnight shaking at room temperature. Subsequently, samples (1 mL) were collected, filtered and transferred to 2 mL HPLC vials. MeSA in the viable tissue (VT) was extracted after cutting the tape-stripped skin into small pieces which were then extracted with 4 mL of the same extraction solvent and by shaking overnight. Samples (1 mL) were again filtered (Sartorius minisart Nylon syringe filter, 25 mm diameter, 0.45 μm pore size (Sartorius AG, Göttingen, Germany)) and transferred to HPLC vials for analysis. This uptake experiment was repeated 8 times.

Finally, three additional MeSA ‘uptake’ experiments were performed in precisely the same manner as above, but without sampling the receptor phase at 2 h. However, post-cleaning of the skin surface, the SC sampling/extraction and the VT processing/extraction steps were delayed by 0.5, 1 and 2 h so as to permit information on drug ‘clearance’ from the SC to be evaluated [17, 32]. During the clearance periods, the diffusion cells were re-covered with a fresh piece of Parafilm and returned to the oven. A 1 mL sample of the receptor solution was acquired at each of the three clearance times for HPLC analysis of permeated MeSA. All clearance experiments were performed in sextuplicate.

An identical series of experiments (*n* = 6 for both uptake and clearance) was performed, in which the skin was occluded during clearance with two layers of loose-weave cotton gauze and a layer of aluminium foil, placed between the skin and donor compartment and held together with a pinch clamp (the top of the donor compartment was left uncovered) in an effort to more effectively block the evaporation of MeSA. The gauze was used to prevent direct skin contact with the aluminium foil.

### In vivo stratum corneum sampling

The protocol, which followed closely a published methodology [33], was approved by the Research Ethics Approval Committee for Health at the University of Bath (REACH EP 18/19 061). Six healthy volunteers provided informed consent and participated in the study. If necessary, the ventral forearm skin was carefully shaved using a new disposable razor at least 24 h before the study began. No lotion, cream or other personal care product on the forearms was used before (at least one day) or during the study.

**Fig. 1:**
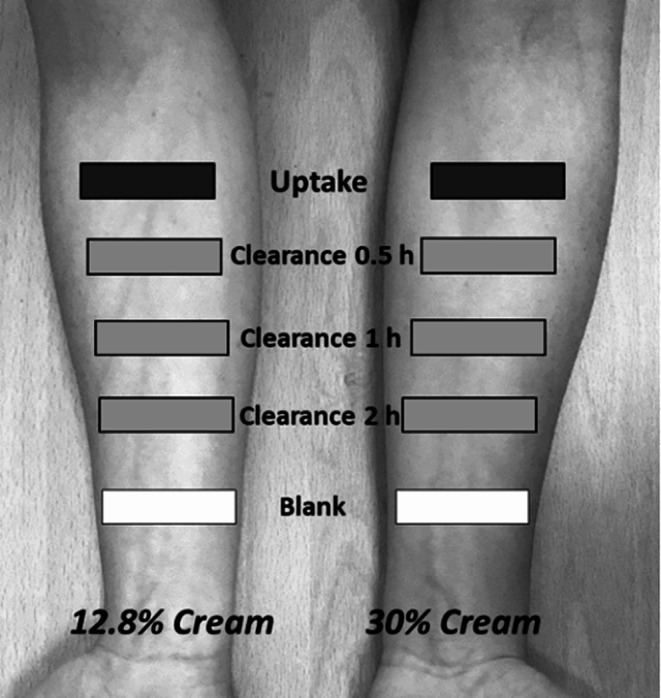
Layout of the MeSA cream treatment sites (and control) on the first volunteer’s arms. The sites were rotated and randomly assigned to the other five participants such that there was an equal number of applications of each cream to left and right arms

The amount of drug in the SC was measured at one ‘uptake’ (2 h), three ‘clearance’ time points (0.5, 1 and 2 h) and from an untreated blank on each arm (separated by 1.6 cm and located at least 5 cm above the wrist and approximately 0.5 cm below the antecubital fossa (Fig. 1)). The four treatment sites were demarcated using rectangular-shaped frames with an 8.25 cm^2^ (1.5 cm x 5.5 cm) open area cut from self-stick adhesive foam (Pressure Point Foam Padding, Scholl, Slough, UK). The two MeSA creams were applied at a single dose of 5 mg cm^− 2^ to the randomly-assigned 8 treatment sites using inverted centrifuge tubes; the mass of product applied was determined by weight difference. Immediately post-application, each treated site was covered with a layer of Parafilm held on the skin by Mefix^®^ tape (Molnlycke, Lancashire, UK). In vivo experiments with potentially occlusive coverings of gauze and aluminium foil were not performed because the protocol approved by the ethics committee stipulated, based on the Patient Information Leaflets, that the creams should not be applied under occlusion [28, 29]. Covering with Parafilm enabled direct comparison between the in vitro and in vivo clearance phases.

At the end of the 2 h ‘uptake’ period, the frames were removed, and residual drug was cleaned from all the treated skin sites as described above. The designated ‘uptake’ sites were then tape-stripped immediately having first used a thin template of adhesive tape to define a central 5 cm^2^ area (1 cm x 5 cm) of the drug application site. Meanwhile, the edges of the ‘clearance’ sites were demarcated using new rectangular-shaped frames and re-covered with a layer of fresh Parafilm held on the skin as before. As for ‘uptake’, the thin template was applied to demarcate the central area prior to tape-stripping the ‘clearance’ sites at 0.5, 1 and 2 h later.

To ensure that tape stripping removed most of the SC at all ‘uptake’ and ‘clearance’ sites, without complete derangement of the barrier, transepidermal water loss (TEWL) was measured, with an evaporimeter (AquaFlux^®^, Biox System Ltd. London, UK), before (at the sites of treatment) and during SC sampling. Tape-stripping was stopped as soon as one of the following occurred: (a) TEWL reached 60 g·m^− 2^·h^− 1^, (b) the TEWL measured was > 6-fold the baseline, pre-stripping value, or (c) 30 tapes had been removed. MeSA was extracted from groups of tape-strips as above. The untreated blank samples of SC were acquired from each volunteer and subjected to the identical extraction/analysis procedure to confirm the absence of any interference in the MeSA assay.

### MeSA evaporative loss from the formulations

The two MeSA creams were applied at 5 mg cm^− 2^ (using an inverted centrifuge tube as before) for periods of 0, 0.25, 0.5, 1 and 2 h to the backing material (polypropylene) of two adhesive tapes that were adhered together. The application area was delimited with a rectangular frame of 8.25 cm^2^ (1.5 cm x 5.5 cm) open area cut from self-stick adhesive foam as used in the in vivo stratum corneum sampling experiments. Care was taken so that contact between the creams and the foam frame was avoided. The samples were covered with a layer of Parafilm affixed with Mefix^®^ tape for the duration of each experiment, after which they were uncovered and the frame removed. Then, the entire adhesive tape substrate was inserted into 10 mL of extraction solvent (MeOH/acetic acid (1%) 90:10) and MeSA was extracted from the residual creams and tape (the remaining drug that had not evaporated) and processed as described above prior to analysis by HPLC. Drug absorption into the tape substrate, if it did occur, would have been small enough to minimally affect the drug mass in the applied creams, and therefore, the drug amount that evaporated. The drug content was verified for the two products.

### HPLC assay of MeSA

The HPLC-UV [Shimadzu liquid chromatograph LC-2010 (Buckinghamshire, UK)] method used was a modification of one already published [34]. A reversed-phase C18 column (HiQ sil C18HS C18 column 250 mm × 4.6 mm, 5 μm (Kromatek, UK)) was employed, and the mobile phase was 70:30 MeOH/water; the column temperature was 25 °C, the flow rate 1 mL min^− 1^, with an injection volume of 50 µL and a detection wavelength at 240 nm. The retention time of MeSA was 6 min. Quantification was based on calibration curves of the compounds dissolved in the mobile phase over the concentration range of 0.05 to 50 µg mL^− 1^. Linearity was confirmed over this range, with a correlation coefficient (r²) of 0.999. Precision was evaluated by assessing variability at low, medium, and high concentrations, with relative standard deviation values consistently below 2%. Accuracy was verified through recovery studies of low, medium, and high concentrations, which showed recoveries between 95% and 106%. The limits of detection and quantification for MeSA were 0.003 and 0.010 µg/mL, respectively.

### Data treatment

IVRT results were expressed as the cumulative amount released from the two creams and into the receptor phase of MeSA (µg cm^− 2^) and the % ‘dose’, as well as the flux (µg cm^− 2^ h^− 1^), as a function of time; the latter was plotted at the mid-point of the sampling interval. IVPT data from the 24 h experiments were expressed similarly, with time, as the cumulative amount permeated across the skin from the two creams and into the receptor phase of MeSA (µg cm^− 2^) and the % ‘dose’, together with the corresponding flux (µg cm^− 2^ h^− 1^). From the shorter 4 h IVPT experiments, the cumulative amounts of MeSA (µg cm^− 2^) found in the SC, VT and receptor phase, together with a mass balance, as a function of time were recorded.

The in vivo and in vitro SC sampling data were expressed as amount of MeSA (µg cm^− 2^) measured immediately after the 2 h ‘uptake’, and then after subsequent clearance periods of 0.5, 1 and 2 h. The apparent first-order rate constant describing MeSA transfer from the SC into the VT was calculated from the linear regression (GraphPad Prism 5.01 version 9.3.1 (San Diego, CA)) of the natural logarithm of the cumulative SC amounts (at 0, 0.5, 1 and 2 h of clearance, where ‘0’ is the ‘uptake’ value) versus time [30]. A similar analysis was applied to the total MeSA content (SC, VT, and receptor compartment combined) in the in vitro SC sampling experiments to describe MeSA loss during clearance, presumably by evaporation from the skin surface. The evaporative loss of MeSA from the two products placed on a surface and covered with Parafilm was also modelled using first-order kinetics to determine a corresponding rate constant. For the in vivo experiments, the first-order rate constant was determined from linear regression of the data from each subject. For each cream, GraphPad Prism did not find significant inter-subject differences in the derived rate constants for either cream and a pooled rate constant for each formulation was therefore determined together with the median value for the regression coefficients. A second regression analysis including all the in vivo data showed that there were no differences between the rate constants determined for the two creams and a pooled value for all the in vivo data was determined.

Statistically significant differences were estimated using [GraphPad Prism 5.01 version 9.3.1 (San Diego, CA)] by one-way ANOVA followed by Tukey’s test. In all comparisons made, statistical significance was set at *p* < 0.05.

## Results

### IVRT experiments

**Fig. 2:**
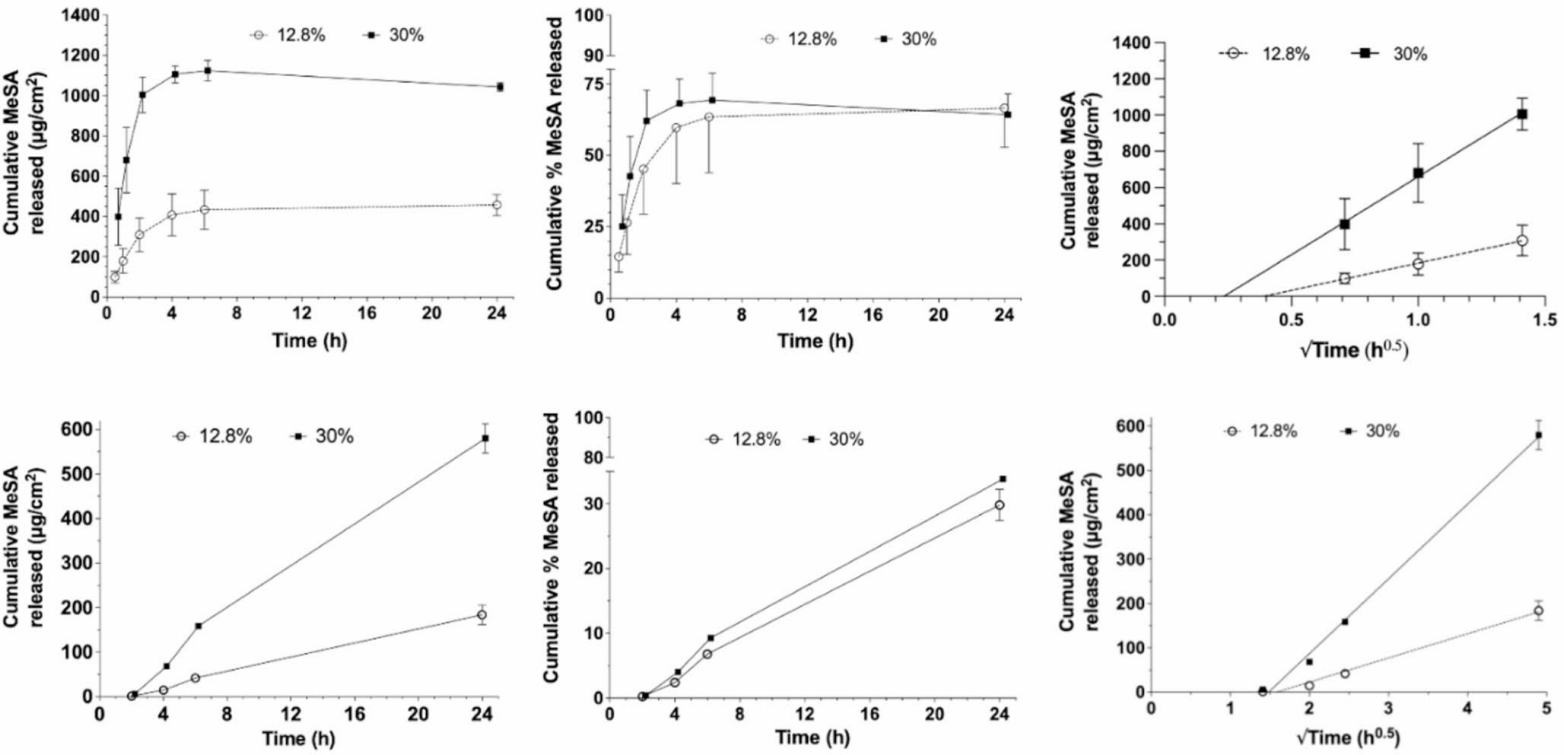
IVRT results for MeSA release from 12.8% and 30% creams across a silicone membrane (top) and Parafilm membrane (bottom). Left panel: cumulative MeSA release as a function of time. Middle panel: cumulative MeSA release expressed as a percentage of the initial drug loading in the creams. Right panel: cumulative MeSA release over the first 2 h (silicone membrane) or 24 h (Parafilm membrane) of the experiments as a function of the square-root of time. Values presented are the mean ± SD of six replicate measurements; some data points have been shifted on the x-axis to facilitate visualisation

The IVRT results using a silicone membrane are shown in Fig. 2. The cumulative amount of MeSA released in 24 h was predictably (and significantly) higher from the 30% cream (1043 ± 22 µg cm^− 2^) than that from the 12.8% formulation (457 ± 53 µg cm^− 2^); the ratio of these quantities was approximately 2.28 consistent with that of the respective drug loadings (2.34), and assuming that MeSA is fully dissolved in both creams. When expressed as a percentage of the initial loading of the two creams, the cumulative amounts released in 24 h for the 30% and 12.8% formulations were not significantly different: 64.2(± 7.3)% *versus* 66.5(± 13.7)%, respectively. The release profiles of MeSA from both creams were essentially identical, exhibiting classic ‘burst’ behaviour with rapid initial depletion over the first 2 h, followed by a sharp deceleration over 4 h, after which further drug release effectively ceased, indicating that approximately 35% of the drug was not accounted for at the end of the experiment. In accord with the short-time approximation of Fick’s 2nd law, cumulative drug release up to 2 h was proportional to the square-root of time [35] with slopes of 861 µg cm^− 2^ h^− 1/2^ and 300 µg cm^− 2^ h^− 1/2^ for the 30% and 12.8% creams, respectively; the corresponding intercepts on the x-axis were 0.23 and 0.39 h^1/2^.

The release of MeSA across Parafilm membranes from the two creams, also shown in Fig. 2, was slower but generally mirrored the release behaviour of the silicone membranes. In this case, the slopes and intercepts were 168 µg cm^− 2^ h^− 1/2^ and 1.5 h^1/2^ for the 30% cream, and 55 µg cm^− 2^ h^− 1/2^ and 1.6 h^1/2^ for the 12.8% cream.

### IVPT experiments and in vitro stratum corneum sampling

**Fig. 3:**
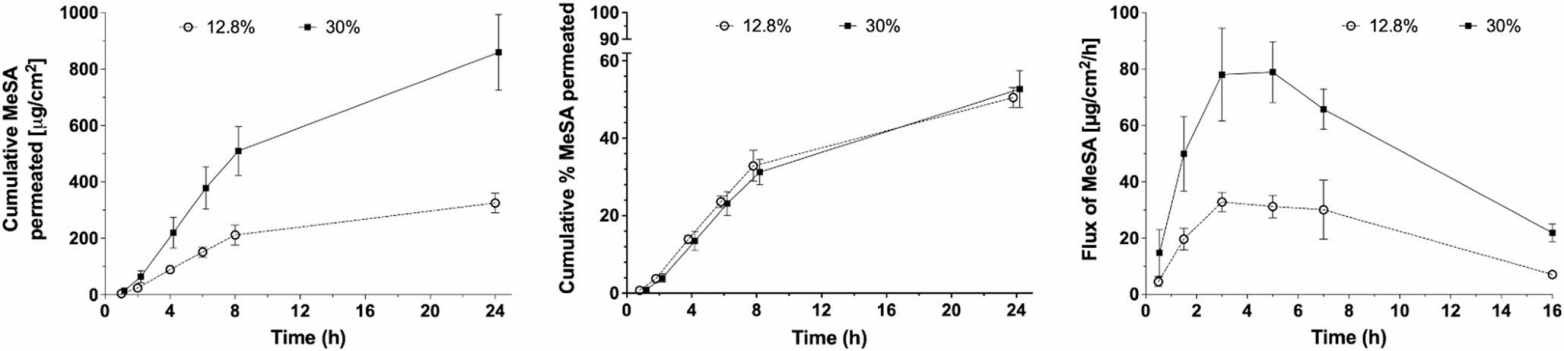
IVPT results for MeSA permeation across excised porcine skin from 12.8% and 30% creams. Left panel: cumulative drug permeation as a function of time. Middle panel: cumulative MeSA permeation expressed as a percentage of the initial drug loading in the creams. Right panel: flux of drug into the Franz cell receptor solution as a function of time (data calculated from successive pairs of the values in the left panel and plotted at the mid-point of the sampling interval). Values presented are the mean ± SD of six replicate measurements; some data points have been shifted on the time-axis to facilitate visualisation

The IVPT results for MeSA permeation across excised pig skin following application of the 12.8% and 30% MeSA creams are shown in Fig. 3. Cumulative drug permeation at 24 h was unsurprisingly - and significantly - greater from the 30% formulation (860 ± 135 µg/cm^2^) than that from the 12.8% (326 ± 35 µg/cm^2^); in this case, the ratio of these quantities was ~ 2.64 (i.e., within 13% of the expected value, assuming as before that the solubilities of MeSA in the two creams are the same and are greater than 30%). When expressed as a percentage of the initial loading of the two creams, the cumulative permeation of MeSA in 24 h for the 30% and 12.8% formulations were not significantly different: 52.7(± 4.8)% *versus* 50.5(± 2.6)%, respectively. Lastly, the flux profiles of the drug into the diffusion cell receptor phase were calculated from successive pairs of the cumulative permeation data and showed that MeSA flux– after a relatively short lag-time– reached a maximum for both the 12.8% and 30% creams between 3 and 5 h before decreasing thereafter as the drug depleted from the formulations. The maximum fluxes achieved were significantly different: 79(± 11) µg cm^− 2^ h^− 1^ and 33(± 3.4) µg cm^− 2^ h^− 1^ for the 30% and 12.8% creams, respectively; the ratio of these values is 2.41.

**Fig. 4:**
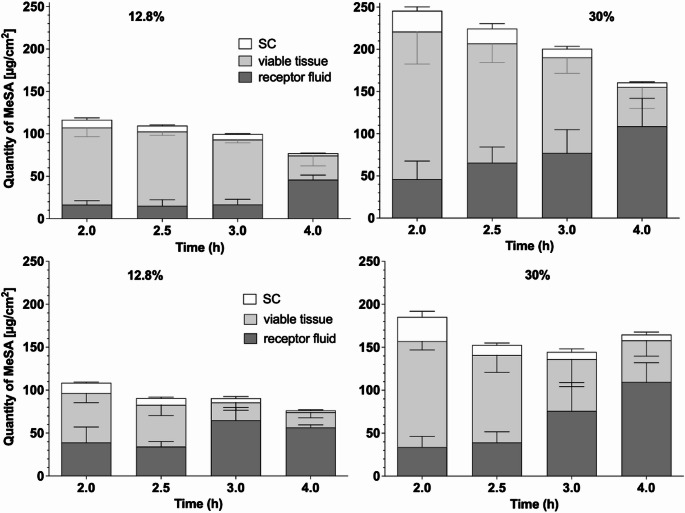
Quantities (mass per unit area) of MeSA measured in the SC, VT and diffusion cell receptor phase following uptake (2 h) while covered with Parafilm and clearance (i.e., the subsequent 0.5, 1 and 2 h) post-removal of the 12.8% and 30% creams when the donor compartment of the diffusion cells was covered with fresh Parafilm (upper panels) or the skin was covered with gauze and aluminium foil (lower panels). The results are the mean + SD of 6 replicates, except for uptake of the Parafilm covered diffusion cells, which had 8 replicates

Analysis of MeSA within the SC and VT was performed to provide further insight into the drug’s dermatopharmacokinetics (a) immediately after a 2 h application (uptake) of the two creams, and (b) post-removal of the formulations following further (clearance) periods of 0.5, 1 and 2 h. The amounts of MeSA recovered from the receptor phase of the diffusion cell after the uptake and three clearance times were also determined. The results obtained when the diffusion cells were covered with fresh Parafilm at the start of uptake and clearance periods are summarised in Fig. 4 (upper panels) and Supplementary Table 1.

The levels of MeSA in the SC and VT were, as anticipated, maximal at the end of the 2 h uptake period, with the quantities in both skin layers being higher following application of the 30% cream compared to the 12.8%. Predictably, as well, the amounts of MeSA in the SC and VT decreased with increasing time of clearance. Drug permeation into the receptor solution at the end of the 2-h uptake then increased gradually over the subsequent clearance period. Total recovery in the SC, VT and receptor was, of course, higher from the 30% cream, with ratios (30%/12.8%) at uptake and at 0.5, 1 and 2 h of clearance being 2.12, 2.04, 2.01 and 2.09, respectively.

For both creams, however, there was a clear trend of decreasing drug mass recovered with the total experiment duration– that at 2 h uptake being very significantly different to that after 2 h of clearance (1-way ANOVA followed by Tukey’s multiple comparisons test) - indicative of an unaccounted drug loss pathway believed to be volatilisation from the skin surface. The slope of linear regressions of the log-transformed total MeSA content (amounts in the SC, VT, and receptor compartment combined) yields first-order disappearance rate constants of 0.21 h^− 1^ for both the 12.8% and 30% creams (r^2^ = 0.66 and 0.74, respectively; see Supplementary Table 4 for full details). A similar analysis of MeSA amounts in the SC over 2 h of clearance gives first-order clearance rate constants of 0.58 h^− 1^ (r^2^ = 0.81) and 0.80 h^− 1^ (r^2^ = 0.84) for the 12.8% and 30% formulations, respectively (Supplementary Table 4).

Data from a similar set of experiments in which the skin was covered with gauze and aluminium foil during the clearance phases are also presented in Fig. 4; detailed results are listed in Supplementary Tables 2 and 4. The MeSA quantities in the SC, VT and receptor phase roughly mirrored those in the Parafilm-occluded experiments. For the 12.8% cream, the total drug mass recovered at 2 h uptake was again significantly different to that after 2 h of clearance; for the 30% formulation, the total MeSA uptake at 2 h was significantly greater than those after 0.5 and 1 h of clearance. The first-order disappearance rates of MeSA from the SC as well as from the SC, VT and receptor chamber combined were not, in general, significantly different for the Parafilm and gauze/aluminium foil coverings suggesting that the latter did not meaningfully interfere with the drug loss pathway.

### In vivo stratum corneum sampling

**Fig. 5:**
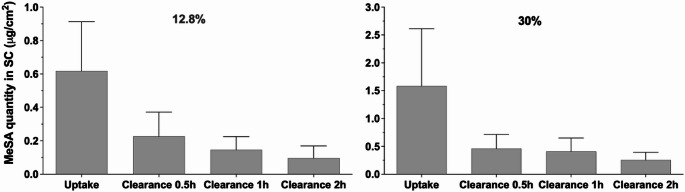
Quantities (mass per unit area) of MeSA measured in the SC in vivo following uptake (2 h) and clearance (i.e., the subsequent 0.5, 1 and 2 h) post-removal of the 12.8% and 30% creams; in these experiments the treated skin sites were covered with Parafilm applied fresh at the start of the uptake and the clearance periods. The results are the mean + SD of results from 6 participants (except for the 30% uptake for which one significant outlier result was excluded)

The quantities of MeSA in the SC at the uptake and clearance sites after a 2-h application of the two creams are reported in Fig. 5 and Supplementary Table 3. At the end of the uptake period, the amount of drug present in the SC was 0.62 (± 0.28) µg cm^− 2^ from the 12.8% formulation and 1.58 (± 0.83 µg cm^− 2^ from the 30% product (corresponding to a ratio of 2.55).

**Fig. 6:**
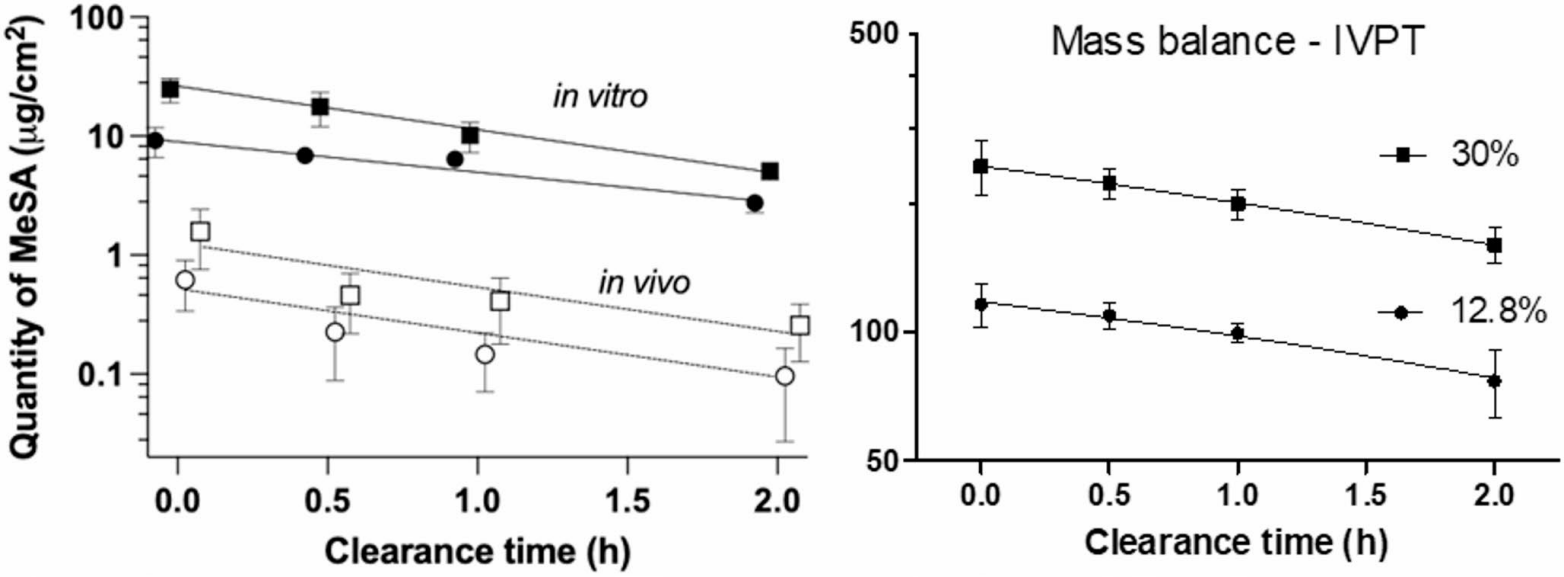
Clearance of MeSA from the SC (left) and the mass balance (SC, VT and receptor chamber combined) (right) versus time following uptake (2 h) and clearance (i.e., the subsequent 0.5, 1 and 2 h) post-removal of the 12.8% (circles) and 30% creams (squares) in vitro (solid symbols) and in vivo (open symbols). Fresh Parafilm was used for the clearance phase. The results are mean ± SD of the results from 6 replicates (participants), except at zero clearance time for in vitro, which had 8 replicates. Details on the specific data are in the legends to Figs. 4 and 5. Data have been displaced slightly on the time axis to facilitate viewing. The lines represent linear regressions of the log-transformed MeSA quantities as described in the text

As in the in vitro experiments, MeSA levels in the SC during clearance decreased with approximately first-order kinetics reflecting (at least) two loss processes based on the results already presented, namely drug permeation into the viable skin, as well as volatilisation from the skin surface. The in vitro SC sampling experiments exhibited the same clearance kinetics, and the results are compared with the in vivo data in Fig. 6.

Linear regression of the log-transformed MeSA amounts in the SC in vivo was used to determine the first-order disappearance rate constants for each participant. Because no differences were found among the values, a pooled rate constant was determined for each cream. The pooled rate constants and median coefficient of regressions were 0.82 h^− 1^ and r^2^ = 0.66 for the 30% cream and 0.80 h^− 1^ and r^2^ = 0.77 for the 12.8% cream (see Supplementary Table 4 for further details). In addition, no significant differences were found between the slopes corresponding to the two creams, so it is possible to calculate a pooled rate constant for all the in vivo data of 0.80 h^− 1^ (corresponding to a pooled half-life of 0.87 h).

### MeSA evaporative loss from the formulations

**Fig. 7:**
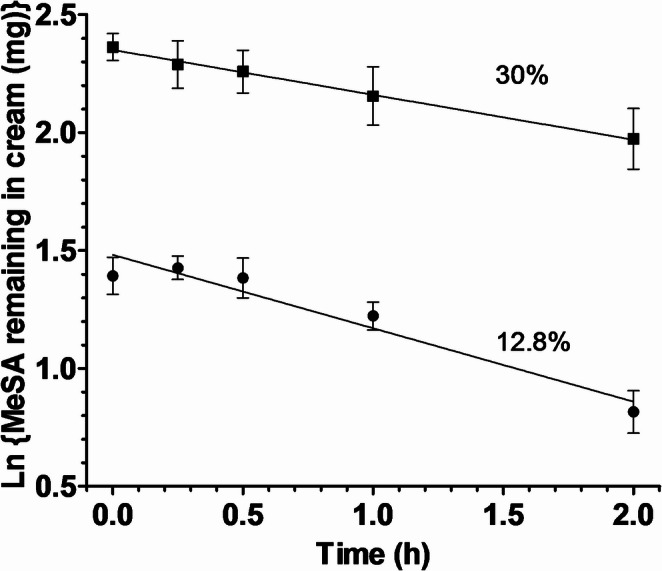
First-order evaporative loss of MeSA from the two formulations on an adhesive tape surface and covered by Parafilm. The results presented are the mean ± SD from 3 replicates; r^2^ values are 0.88 and 0.72 for the 12.8% and 30% creams, respectively

The first-order kinetics of evaporative loss of MeSA from the 12.8% and 30% creams placed on a surface and covered in Parafilm are shown in Fig. 7. Linear regressions of the log-transformed MeSA amounts measured as a function of time indicate that the first-order rate constant for MeSA loss from the 12.8% formulation (0.31 h^− 1^) was significantly greater (*p* < 0.05) than that for the 30% product (0.19 h^− 1^).

## Discussion

This research aimed to identify whether different approaches to assess cutaneous PK can be combined to assess the rate and extent by which a volatile drug becomes bioavailable at (or close to) its presumed site of action. Three well-studied methods were included: IVRT, IVPT and SC sampling with the latter two enabling cutaneous PK metrics to be deduced in different skin compartments. In parallel, independent measurements of the evaporation of the model drug (MeSA) from the two products employed were made. The validation of methods to determine the BA of drugs delivered from complex topical products is an area of significant current interest [36]. While IVRT and IVPT are established tools in this regard, the SC sampling technique [33]– despite many positive reports of its value [16, 17, 37, 38]– has only received full endorsement from the Japanese and, most recently, European Union regulatory authorities [7]. However, the application of all methodologies is yet to be assessed to any depth for active pharmaceutical ingredients that are volatile and prone to evaporative loss during and post-application to the skin.

Initially, the release of MeSA from the two products was tested in a standard IVRT experiment. The release behaviour through a silicone membrane (Fig. 2**)** was consistent with drug in solution in the formulations, which is release followed classic kinetic profiles in proportion to MeSA content. The percentage of the dose applied that was released from the two products was, therefore, essentially identical. The slopes of the initial cumulative release as a function of square-root of time were linear. Despite the relatively rapid MeSA release observed, however, the total mass balance in these experiments was clearly well below 100% of that applied - with no more than 70% recovered - implying evaporative loss of the drug during the experiment through the Parafilm used to occlude the diffusion cell donor compartment. The evaporative loss of MeSA was confirmed by experiments tracking drug loss when the creams were placed on a surface and covered with Parafilm (Fig. 7). The average first-order evaporation rate constants were 0.31 h^− 1^ for the 12.8% cream and 0.19 h^− 1^ for the 30% product. Parenthetically, in an additional IVRT study, release of MeSA was slower across Parafilm than through the silicone membrane (Fig. 2), although the kinetic profiles were otherwise similar. Thus, although Parafilm is a good barrier to water (water vapor permeability is 0.037 g m^− 2^ h^− 1^; [39]), this is not the case for organic molecules, such as MeSA.

Subsequently, IVPT experiments conducted over 24 h showed that skin is a better barrier to MeSA diffusion than silicone (Fig. 3). The cumulative delivery of drug into the receptor differentiated formulations clearly and in an approximately proportionate way; the percentage dose delivered from the two creams was essentially identical. The flux profiles were proportionate, too, with both achieving maximum values of flux between 3 and 5 h. Although a clear plateau of the percentage drug permeated was not observed (perhaps because samples were not collected between 8 and 24 h), drug fluxes were very small at 24 h, consistent with drug depletion, arising from dermal absorption (~ 60% of applied dose is in receptor solution) as well as evaporation. Mass balances from in vitro SC sampling over the 2 h of clearance (with Parafilm coverage of the donor compartment as before; Fig. 4) also provide clear evidence of ‘missing’ drug (approximately 30% less drug; Supplementary Table 1) consistent with loss by evaporation from the SC. Similar amounts of ‘missing’ drug were also observed when the donor compartment was covered during clearance with gauze and aluminium foil. This suggests that the foil did not form a tight seal of the donor compartment and/or evaporated MeSA absorbed to the gauze to approximately the same extent as it absorbed (and permeated through) the Parafilm.

In vivo SC sampling, again involving a 2 h uptake and a further 2 h of clearance, revealed significantly smaller uptake than that observed in vitro for both products (Fig. 5). Nonetheless, as for the IVRT and IVPT results discussed above, the 30% cream delivered more MeSA into the SC than the 12.8% cream by a factor of 2.55, which is close to that predicted by the ratio of drug concentrations in the product (2.34). The causes of the difference between in vivo and in vitro SC uptake/clearance data are unknown. A possible explanation for part of the difference is enhanced drug removal from the in vivo dermis due to an active microcirculatory clearance mechanism (e.g. cutaneous blood flow), and/or the known vasodilatory action of the drug on cutaneous microvessels [40], both of which are missing in the in vitro experiments. MeSA may have accumulated in the in vitro skin layers post-application given the absence of any clearance mechanism other than the relatively slow passive diffusion through the underlying ‘viable’ tissue, which in the pig skin samples is roughly 700 μm thick and without a functional microcirculation. Other possible contributing factors include (i) more MeSA evaporation from the cream during the longer application time required for the larger in vivo area (8.25 cm^2^ vs. 2.01 cm^2^) as well as during the uptake period due to an elevated skin temperature in vivo from a smaller air volume above the skin surface and insulation by the Mefix tape over the Parafilm, (iii) differences in MeSA partitioning and permeability in pig and human SC, and (iv) other unidentified mechanisms (e.g., impact on skin barrier of methanol in the IVPT receptor chamber).

Despite the differences in the in vivo and in vitro SC uptake, however, MeSA clearance from the SC, as shown in Fig. 6, is described by a first-order elimination rate-constant that is similar for drug delivered from both creams and for both in vivo and in vitro scenarios (the mean of the four deduced rate constants for skin covered with Parafilm during clearance is 0.75 (± 0.11) h^− 1^; Supplementary Table 4). This implies that in vivo and in vitro mechanisms contributing to the SC clearance of drug are the same or similar. The decreasing total recovery presented in Fig. 6 underscores that MeSA evaporation must contribute to this overall clearance, as must the diffusive transport of the drug out of the SC into the underlying viable tissue and beyond. In the in vitro experiments, the first-order rate constants for the (evaporative) loss of MeSA during 2 h of clearance were 0.21 h^− 1^ for both the 30% and 12.8% creams.

In clearing from the SC, MeSA undergoes two competing first-order ‘loss’ processes: evaporative loss (with a rate-constant, *k*_*evap*_) and diffusion into the deeper skin (with a rate-constant, *k*_*diff*_). The in vitro and in vivo experiments provide a consistent value of the overall elimination rate constant (*k*_*elim*_), which has an average of 0.75 h^**−** 1^. Assuming that *k*_*elim*_ = *k*_*evap*_ + *k*_*diff*_, we deduce that *k*_*diff*_ for MeSA will be approximately 0.54 h^− 1^ (i.e., *k*_*elim*_ - *k*_*evap*_, where *k*_*evap*_ = 0.21 h^− 1^), which corresponds to a lag time for SC permeation of approximately 0.31 h (*k*_*diff*_ = 1/(6 *t*_*lag*_) [33]). This *k*_*diff*_ value is higher than those deduced from SC sampling experiments for other drugs (e.g. acyclovir, diclofenac, econazole, and scopolamine [16, 17, 33, 37, 38, 41]) but sensible on the basis of MeSA having a smaller molecular weight (152.2 g/mol), which leads to greater diffusivity.

## Conclusion

The application of IVRT, IVPT and SC sampling to the assessment of the cutaneous PK of a topically applied, volatile drug (MeSA) has enabled metrics characterizing its local bioavailability to be derived. The PK parameters calculated from the experiments performed, together with measurements of MeSA’s evaporation kinetics, were consistent between two commercially available products that contained distinctly different quantities of the drug. Overall, therefore, despite complications introduced by a non-biological loss process of the drug (volatilisation) as well as MeSA’s pharmacological ability to enhance its own clearance from the skin by action on the dermal microcirculation, the complementary nature of in vitro and in vivo methods was sufficiently robust to yield information descriptive of the disposition of a volatile drug in the skin.

## Electronic supplementary material

Below is the link to the electronic supplementary material.

Supplementary Material 1

## Data Availability

The datasets generated during and/or analysed during the current study are available from the corresponding author on reasonable request. Materials were purchased from sources described in the Methods section.
