# Supplementary material for: Cutaneous pharmacokinetics of a volatile drug post-application to the skin
Source: Drug Deliv Transl Res. 2025 Jun 26;16(7):2130–41. doi: 10.1007/s13346-025-01907-8 (PMC13294149; doi:10.1007/s13346-025-01907-8)
Supplement: Supplementary file 1 — Supplementary Material 1 [file 13346_2025_1907_MOESM1_ESM.docx]

## Supplementary information

**Cutaneous pharmacokinetics of a volatile drug post-application to the skin**

**Andrea Pensado^1,2^, Panagiota Zarmpi^1,3^, Jane White^4^, Annette L. Bunge^5^, Richard H. Guy^1^, M. Begoña Delgado-Charro^1,*^**

^1^University of Bath, Department of Life Sciences, Claverton Down, Bath, BA2 7AY, U.K.

^2^Current address: Center for Research in Molecular Medicine & Chronic Diseases (CiMUS), Universidade de Santiago de Compostela, 15782 Santiago de Compostela, Spain

^3^Current address: University of Surrey, School of Chemistry and Chemical Engineering, Guildford, GU2 7XH, U.K.

^4^ University of Bath, Department of Mathematical Sciences, Bath, BA2 7AY, U.K.

^5^Department of Chemical & Biological Engineering, Colorado School of Mines, Golden, CO 80401, U.S.A.

**Supplementary Table 1:** Quantities (mass per unit area) of MeSA measured in the SC, VT and diffusion cell receptor phase following uptake (2 h) and clearance (i.e., the subsequent 0.5, 1 and 2 h) post-removal of the 12.8% and 30% creams. In these experiments the donor compartment of the diffusion cells was covered with Parafilm. The results are the mean ± SD of 8 and 6 replicates for the uptake and clearance measurements, respectively.

| MeSA (%) | Uptake (h) | Clearance (h) | SC (μg cm^-2^) | VT (μg cm^-2^) | Receptor (μg cm^-2^) | Total (μg cm^-2^) |
| --- | --- | --- | --- | --- | --- | --- |
| 12.8 | 2.0 | 0 | 9.2 ± 2.6 | 91 ± 13 | 16 ± 5.8 | 116 ± 14 |
| 12.8 | 2.0 | 0.5 | 6.9 ± 0.9 | 87 ± 4.1 | 15 ± 6.9 | 110 ± 8.2 |
| 12.8 | 2.0 | 1.0 | 6.4 ± 0.6 | 76 ± 3.8 | 17 ± 5.9 | 100 ± 5.3 |
| 12.8 | 2.0 | 2.0 | 2.8 ± 0.5 | 28 ± 12 | 46 ± 5.1 | 77 ± 14 |
|  |  |  |  |  |  |  |
| 30 | 2.0 | 0 | 25 ± 5.7 | 175 ± 46 | 46 ± 26 | 246 ± 35 |
| 30 | 2.0 | 0.5 | 18 ± 5.6 | 141 ± 22 | 66 ± 18 | 224 ± 18 |
| 30 | 2.0 | 1.0 | 10 ± 2.9 | 113 ± 18 | 77 ± 26 | 201 ± 16 |
| 30 | 2.0 | 2.0 | 5.1 ± 0.9 | 47 ± 24 | 109 ± 32 | 161 ± 15 |

**Supplementary Table 2:** Quantities (mass per unit area) of MeSA measured in the SC, VT and diffusion cell receptor phase following uptake (2 h) and clearance (i.e., the subsequent 0.5, 1 and 2 h) post-removal of the 12.8% and 30% creams. In these experiments the donor compartment of the diffusion cells was covered with gauze and a layer of aluminium foil. The results are the mean ± SD of 6 replicates.

| MeSA (%) | Uptake (h) | Clearance (h) | SC (μg cm^-2^) | VT (μg cm^-2^) | Receptor (μg cm^-2^) | Total (μg cm^-2^) |
| --- | --- | --- | --- | --- | --- | --- |
| 12.8 | 2.0 | 0 | 12 ± 0.7 | 57 ± 10 | 39 ± 17 | 109 ± 17 |
| 12.8 | 2.0 | 0.5 | 7.8 ± 1.2 | 49 ± 12 | 34 ± 5.6 | 91 ± 9.8 |
| 12.8 | 2.0 | 1.0 | 4.8 ± 2.0 | 21 ± 8.6 | 65 ± 14 | 91 ± 20 |
| 12.8 | 2.0 | 2.0 | 2.2 ± 0.6 | 18 ± 6.0 | 57 ± 2.9 | 76 ± 5.5 |
|  |  |  |  |  |  |  |
| 30 | 2.0 | 0 | 28 ± 6.5 | 123 ± 9.7 | 34 ± 12 | 185 ± 9.7 |
| 30 | 2.0 | 0.5 | 12 ± 2.3 | 102 ± 19 | 39 ± 12 | 153 ± 17 |
| 30 | 2.0 | 1.0 | 8.4 ± 3.4 | 60 ± 30 | 76 ± 31 | 145 ± 16 |
| 30 | 2.0 | 2.0 | 6.9 ± 2.3 | 48 ± 15 | 110 ± 18 | 165 ± 28 |

**Supplementary Table 3:** Quantities (mass per unit area) of MeSA measured in the *in vivo* SC following uptake (2 h) and clearance (i.e., the subsequent 0.5, 1 and 2 h) post-removal of the 12.8% and 30% creams. In these experiments, the skin surface was covered with Parafilm laid on top of the rectangular-shaped foam frame demarcating the treatment site and held in place by Mefix® tape. The results are the mean ± SD for the uptake and clearance sites in each of the six volunteers.

| MeSA (%) | Uptake (h) | Clearance (h) | SC (μg cm^-2^) |
| --- | --- | --- | --- |
| 12.8 | 2.0 | 0 | 0.62 ± 0.28 |
| 12.8 | 2.0 | 0.5 | 0.23 ± 0.14 |
| 12.8 | 2.0 | 1.0 | 0.15 ± 0.08 |
| 12.8 | 2.0 | 2.0 | 0.10 ± 0.07 |
|  |  |  |  |
| 30 | 2.0 | 0 | 1.58 ± 0.83 |
| 30 | 2.0 | 0.5 | 0.46 ± 0.24 |
| 30 | 2.0 | 1.0 | 0.41 ± 0.23 |
| 30 | 2.0 | 2.0 | 0.26 ± 0.13 |

**Supplementary Table 4:** Rate constants, 95% confidence intervals (CI), and the corresponding half-lives (t_½_) of MeSA clearance from the SC (top) and loss of total MeSA content (SC, VT and receptor combined; bottom) over 2 h after a 2-h application and removal of the 12.8% and 30% creams

|  | MeSA (%) | Experiment | Covering | Rate constant (h^-1^) | 95% CI | t_½_ (h) |
| --- | --- | --- | --- | --- | --- | --- |
| SC | 12.8 | *in vitro* | Parafilm | 0.58 | 0.46 - 0.70 | 1.20 |
| SC | 30 | *in vitro* | Parafilm | 0.80 | 0.65 - 0.94 | 0.87 |
| SC | 12.8 | *in vivo* | Parafilm | 0.80 *^a^* | NA | 0.87 |
| SC | 30 | *in vivo* | Parafilm | 0.82 *^a^* | NA | 0.84 |
| SC | 12.8 | *in vitro* | Gauze/ aluminium foil | 0.87 | 0.73 – 1.01 | 0.80 |
| SC | 30 | *in vitro* | Gauze/ aluminium foil | 0.68 | 0.44 – 0.93 | 1.0 |
|  |  |  |  |  |  |  |
| Total | 12.8 | *in vitro* | Parafilm | 0.21 | 0.15 – 0.28 | 3.3 |
| Total | 30 | *in vitro* | Parafilm | 0.21 | 0.16 – 0.27 | 3.3 |
| Total | 12.8 | *in vitro* | Gauze/ aluminium foil | 0.16 | 0.01 – 0.30 | 4.3 |
| Total | 30 | *in vitro* | Gauze/ aluminium foil | 0.25 *^b^* | 0.80 – 1.31 | 2.8 |
| ^a^ Pooled rate constant for 6 subjects. 95% CI are not available for pooled rate-constants  ^b^ 2h clearance point was not included in the calculation of the rate constant | | | | | | |
